# Domestic Summary

**Published:** 1839-04-27

**Authors:** 


### DOMESTIC SUMMARY.

*New Medical Journal.*—We have received the prospectus of a medical journal, proposed to be published in New York, on the 1st of July next under the auspices of the New York Medical and Surgical Society. The Journal will be issued quarterly, at five dollars a year, payable always in advance. Mr. George Adlard is the publisher. Accompanying the prospectus is a list of able collaborators, under whose conduct we may anticipate that the journal will prove a valuable reinforcement of the ranks of our periodical medical literature. It has our best wishes for its success; and we, of course, most readily accede to the proposal for an exchange.

---

The following has been handed to us for publication, by the Secretary of the Philadelphia Medical Society:

*Extract from the Minutes of the Philadelphia Medical Society.*

"*Resolved*, That all discoveries or improvements in medicine or surgery should be freely promulgated through the appropriate channels of medical information, for the advancement of medical science, and for the good of mankind. And that the appropriation of such discoveries or improvements by their authors to their exclusive pecuniary emolument, by the taking out of patents or otherwise, is at variance with those principles of liberality and beneficence, which should distinguish the medical character."

Ordered to be published.

HENRY KEIM, Jun., *Rec. Secretary.*

*March 27th*, 1839.

